# Environmental Endocrine-Disrupting Chemical Exposure: Role in Non-Communicable Diseases

**DOI:** 10.3389/fpubh.2020.553850

**Published:** 2020-09-24

**Authors:** Manoj Kumar, Devojit Kumar Sarma, Swasti Shubham, Manoj Kumawat, Vinod Verma, Anil Prakash, Rajnarayan Tiwari

**Affiliations:** ^1^National Institute for Research in Environmental Health, Indian Council of Medical Research, Bhopal, India; ^2^Department of Stem Cell Research Sanjay Gandhi Post Graduate Institute of Medical Sciences, Lucknow, India

**Keywords:** endocrine-disrupting chemicals, exposome, hormone receptor, trans-generational effects, pollutant

## Abstract

The exponential growth of pollutant discharges into the environment due to increasing industrial and agricultural activities is a rising threat for human health and a biggest concern for environmental health globally. Several synthetic chemicals, categorized as potential environmental endocrine-disrupting chemicals (EDCs), are evident to affect the health of not only livestock and wildlife but also humankind. In recent years, human exposure to environmental EDCs has received increased awareness due to their association with altered human health as documented by several epidemiological and experimental studies. EDCs are associated with deleterious effects on male and female reproductive health; causes diabetes, obesity, metabolic disorders, thyroid homeostasis and increase the risk of hormone-sensitive cancers. Sewage effluents are a major source of several EDCs, which eventually reach large water bodies and potentially contaminate the drinking water supply. Similarly, water storage material such as different types of plastics also leaches out EDCs in drinking Water. Domestic wastewater containing pharmaceutical ingredients, metals, pesticides and personal care product additives also influences endocrine activity. These EDCs act *via* various receptors through a variety of known and unknown mechanisms including epigenetic modification. They differ from classic toxins in several ways such as low-dose effect, non-monotonic dose and trans-generational effects. This review aims to highlight the hidden burden of EDCs on human health and discusses the non-classical toxic properties of EDCs in an attempt to understand the magnitude of the exposome on human health. Present data on the environmental EDCs advocate that there may be associations between human exposure to EDCs and several undesirable health outcomes that warrants further human bio-monitoring of EDCs.

## Introduction

Effects of environmental pollution on human health are increasingly gaining more attention globally. Environmental pollutants are chemicals that result from human activities, which end up in the environment and subsequently pose risks to human and animal health. Several of these chemicals are collectively known as endocrine-disrupting chemicals (EDCs), which are nowadays gaining more importance in terms of public health due to their widespread effects on human health and potential cause of morbidity.

The Endocrine Society defines EDCs as follows: “an exogenous (non-natural) chemical, or a mixture of chemicals, that interferes with any aspect of hormone action.” These chemicals alter the hormonal balance of the body through several different mechanisms; they can mimic hormones, disrupt hormone synthesis or breakdown, alter the development of hormone receptors, act as hormone antagonists or alter hormone binding. Environmental EDCs are mostly released during manufacture and use of human-made materials such as pesticides, plastics/plasticisers, electronic wastes, flame-retardants, metals, food additives, and personal care products. These EDCs can disturb hormonal balance and consequently result in several health disorders, including developmental and reproductive abnormalities, increased prevalence of hormone-sensitive cancers, neurodevelopmental delays and abnormal growth patterns in children, and alterations in immune function.

**Figure 1:**
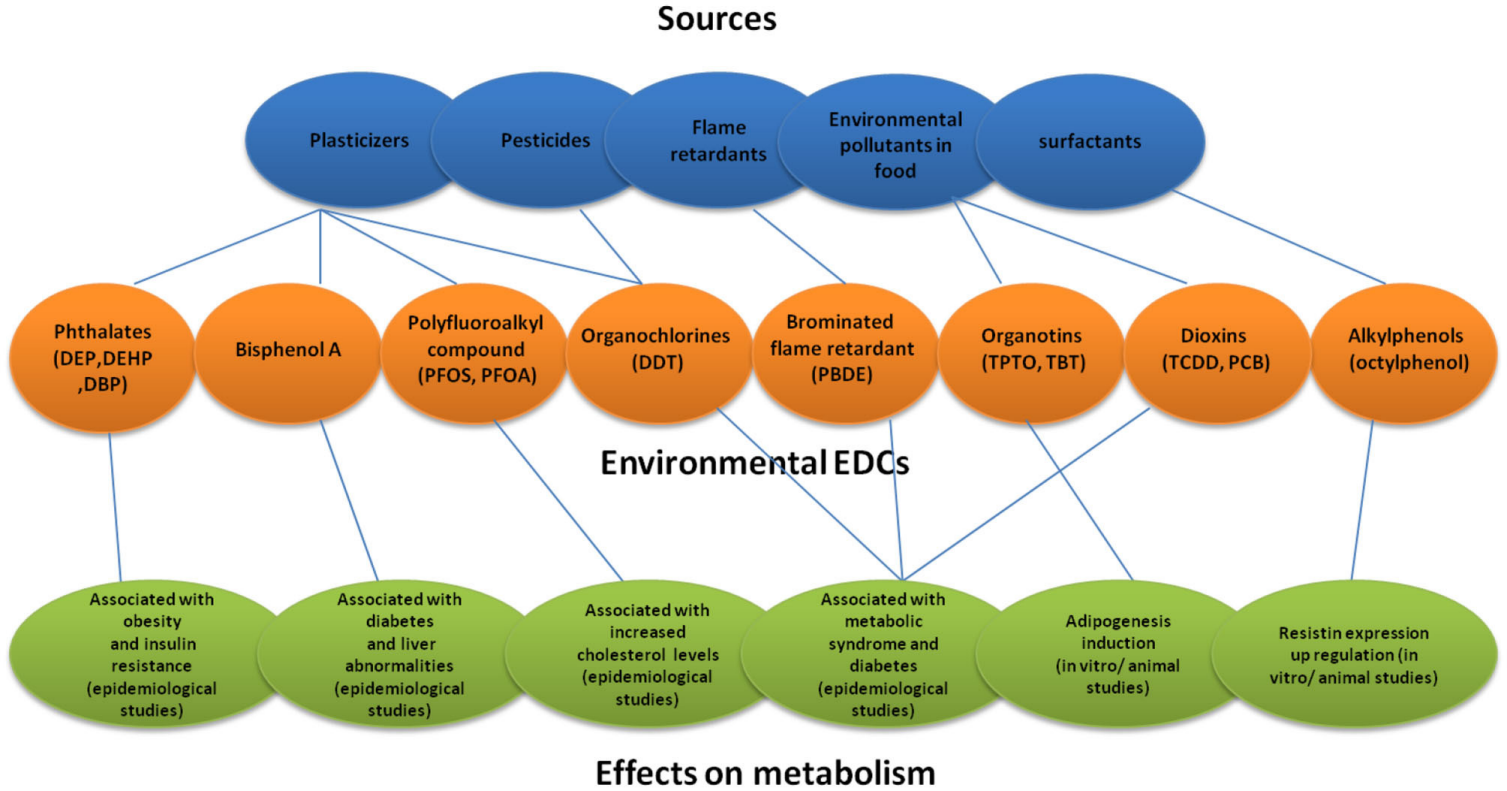
Sources of environmental EDCs and their effects on metabolism. DEP, Diethyl phthalate; DEHP, Diethylhexyl phthalate; DBP, Dibutyl phthalate; PFOS, Perfluorooctanote; DDT, Dichlorodiphenyltrichloro ethane; PBDE, Polybrominated diphenyl ether; TPTO, bis (triphenyltin) oxide; TBT, Tributyltin.

EDCs are a group of highly heterogeneous synthetic chemicals used in a variety of settings. Some of the common chemicals included in this group are chemicals used in industries and their by-products (polychlorinated biphenyls [PCBs], polybrominated biphenyls [PBBs] and dioxins), plastics (bisphenol A [BPA]), plasticisers (phthalates) and pesticides (methoxychlor [MXC], chlorpyrifos and dichlorodiphenyltrichloroethane [DDT]). Some EDCs were designed to have long half-lives for industrial purposes and are known as “persistent organic pollutants” (POPs) exemplified by PCBs, dichlorodiphenyldichloroethylene (DDE), dioxin, organochlorine pesticides and hexachlorobenzene (HCB) (Figure 1). The POPs are highly lipophilic and tend to accumulate in the adipose tissue. Due to this property, they also accumulate in the food chain and undergo biomagnification. Many of these substances either do not decay or decay slowly, while others may be metabolized to compounds that are more toxic than the parent chemicals (1, 2). Other EDCs such as BPA and phthalates may not be persistent but are used so extensively that environmental exposure is widespread. EDCs are present ubiquitously in the environment and human exposure occurs through intake of water and food, via inhalation, and through the skin.

Exposure of plastics to high temperature can lead to leaching or migration of toxic components in environment in the form of microplastics or nanoplastic. In drug delivery system micro- and nanoplastic are used via intravenous, oral, and transcutaneous routes (3), and also there is a migration of nanopolymers from various packaging materials into food (4, 5). Microplastics or nanoplastic accumulates in food chains making them bioavailable again for direct or indirect human exposure via ingestion, skin contact and inhalation (6). Biomonitoring studies have shown that Human consumption of animals that exposed to microplastics and additives persist in human population and can be harmful for human health (7, 8). At present health impact assessments that exclusively focus on the plastic compositions of products while overlook the thousands of plastic additives and their effects.

**Figure 2:**
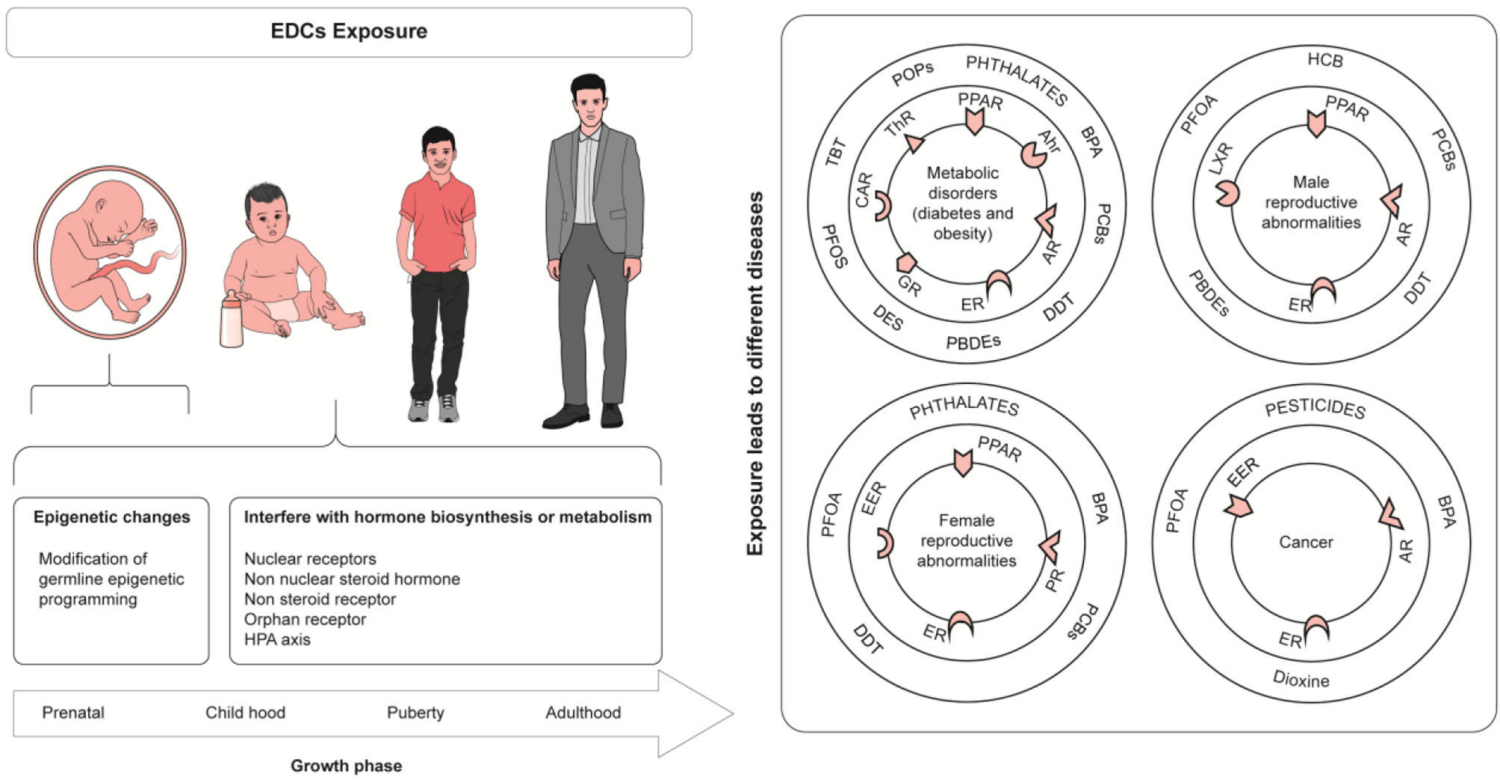
Health Effect of Endocrine disrupting chemicals. Tributyltin (TBT), PerfluorooctaneSulfonate (PFOS), Perfluorinated compounds (PFCs), Perfluorooctanoic acid (PFOA), Bisphenol A (BPA), Diethylstilbestrol (DES), hexachlorobenzene (HCB), dichlorodiphenyltri-chloroethane (DDT), Polybrominated diphenyls ethers (PBDEs), constitutive androstane receptor (CAR), Thyroid Hormone Receptor (ThR), glucocorticoid receptor (GR), Aryl hydrocarbon receptor (Ahr), Androgen receptor (AR), Peroxisome proliferator-activated receptor (PPAR), Estrogen receptor (ER), Liver X Receptor (LXR).

Drinking water particularly is thought to be a source of significant exposure through leaching, industrial waste discharge and inadequate treatment of water supply for chemicals. EDCs can also be transferred from mother to child through trans-placental route as well as through breast milk. Developing fetuses and children are highly susceptible to environmental exposures, and their effects may not become apparent until a later age. Research shows that such exposures may increase the susceptibility of the child to several non-communicable diseases in their adult life (Table 1). EDCs can potentially target various hormone systems; however, it has been observed that the effect of these chemicals primarily involves the reproductive system, puberty, embryonic development and sex differentiation in fetal life. Thus, it is likely that the primary mechanism of their action is through interference with sex steroid hormones. Furthermore, there is also a growing apprehension that metabolic disorders of adult hood may be linked with EDCs. Several *in vitro, in vivo* and epidemiological studies link human-environmental EDC exposures with obesity, metabolic syndrome, type 2 diabetes and possibly with some hormone-responsive cancers (Figure 2).

**Table 1:** Studies documenting associations between different EDC exposures and risk to different diseases.

**Sl. No**.	**Study site**	**Study type**	**Exposure**	**Results/Findings**	**References**
**DIABETES AND OBESITY**					
1	USA	Cross-sectional	POP's (1,2,3,4,6,7,8-heptachlorodibenzo-p-dioxin (HPCDD), 1,2,3,4,6,7,8,9-octachlorodibenzo-p-dioxin (OCDD), oxychlordane, trans-nonchlor, and p,p′-DDT)	Log values of OCDD showed an increase with higher waist circumference (*p* = 0.0052) and Body mass index (BMI) (*p* = 0.0099), In p,p′-DDT and all 5 POP's detected in the study showed a positive correlation with BMI (*P* = 0.0353 and *p* = 0.0001 respectively). Log values of all 5 POP's also showed a positive correlation with waist circumference (*P* = 0.0008)	(15)
2	Denmark	Cohort	perfluorooctanoate (PFOA) (*In utero*)	Adjusted relative risks comparing the highest with lowest quartile (median: 5.8 vs. 2.3 ng/mL) of maternal PFOA concentration were 3.1 [95% CI: 1.4, 6.9] for overweight and 3.0 (95% CI: 1.3, 6.8) for waist circumference	(16)
3	Greece	Cohort	*In utero* exposure to POPs [polychlorinated biphenyls (PCBs), dichlorodiphenyldichloroethylene (DDE) and hexachlorobenzene (HCB)]	A 10-fold increase in HCB was linked with a higher BMI *z*-score (adjusted β = 0.49; 95% CI: 0.12, 0.86), obesity [relative risk (RR) = 8.14; 95% CI: 1.85, 35.81], abdominal obesity (RR = 3.49; 95% CI: 1.08, 11.28) at 4 years of age. Prenatal DDE exposure was linked with higher BMI z-score (β = 0.27; 95% CI: 0.04, 0.5), abdominal obesity (RR = 3.76; 95% CI: 1.70, 8.30), and higher diastolic BP (β = 1.79 mmHg; 95% CI: 0.13, 3.46). PCBs were not significantly associated with offspring obesity or cardio metabolic risk factors	(17)
4	Norwegian and Swedish	Cohort	Perfluorooctanesulfonate (PFOS)	Increased BMI-for-age-and-sex z-score (β = 0.18, 95% CI: 0.01–0.35) in children at 5 year follow-up per ln-unit increase in maternal serum perfluorooctanesulfonate (PFOS) concentrations was observed. Observed increased odds for child overweight/obesity (BMI ≥ 85th percentile) for each. ln-unit increase in maternal serum PFOS levels (adjusted OR: 2.04, 95% CI: 1.11–3.74), with stronger odds among Norwegian children (OR: 2.96, 95% CI: 1.42–6.15). Similar associations were also observed between maternal serum perfluorooctanoate (PFOA) concentrations and child overweight/obesity (OR: 2.04, 95% CI: 1.11–3.74)	(18)
5	USA	Cross-sectional	Bisphenol A (BPA)	BPA was found significantly associated with both general [OR: 1.78 (95% CI 1.10–2.89); *p* = 0·04] and abdominal obesity [OR: 1.55 (95% CI: 1.04–2.32); *p* = 0.02] in studied adults	(19)
6	Mexico	Cohort	Monobenzyl phthalate (MBzP)	Prenatal urinary exposure to monobenzyl phthalate (MBzP) was inversely associated with child's BMI z-score (β = −0.21, 95% CI: −0.41, −0.02) and child urinary exposure to mono(2-ethylhexyl)phthalate (MEHP) was inversely associated with waist circumference (β = −1.85, 95%CI: −3.36, −0.35); In girls, increased BPA exposure was positively associated with BMI z-score (β = 3.47, 95%CI: −0.05, 6.40)	(20)
7	Spain	Cohort	Five high-molecular-weight phthalate metabolites included the simple monoester MEHP, secondary oxidized metabolites MEHHP, MEOHP, and MECPP and MBzP). The low-molecular-weight phthalate metabolites included MEP, MiBP, and MnBP	The sum of five high-molecular-weight phthalate metabolites was associated with lower weight *z*-score difference between birth and 6 months (β per doubling of exposure = −0.41; 95% CI: −0.75, −0.06) and BMI *z*-scores at later ages in boys (β = −0.28; 95% CI: −0.60, 0.03) and with higher weight *z*-score difference (β = 0.24; 95% CI: −0.16, 0.65) and BMI *z*-scores in girls (β = 0.30; 95% CI: −0.04, 0.64) (*p* for sex interaction = 0.01 and 0.05, respectively). The low-molecular-weight phthalate metabolites was not significantly associated with weight gain, BMI z-scores, or waist-to-weight ratio at any age	(21)
8	USA	Cohort	Phthalate metabolite (Prenatal)	Mono-3-carboxypropyl phthalate (MCPP) concentrations were positively associated with overweight/obese status in children (odds ratio [95% credible interval] = 2.1 [1.2, 4.0]). monoethyl phthalate (MEP) and summed di-(2-ethylhexyl) phthalate metabolites (∑DEHP) concentrations were inversely associated with BMI z-scores among girls (MEP beta = −0.14 [−0.28, 0.00]; ∑DEHP beta = −0.12 [−0.27, 0.02])	(22)
9	Sweden, PIVUS study	Cohort	14 polychlorinated biphenyl [PCB] congeners, 3 organochlorine pesticides, 1 brominated diphenyl ether, and 1 dioxin	plasma concentrations of POPs, especially PCBs and organochlorine pesticides, strongly predicted incident type 2 diabetes during a 5 year follow-up	(28)
10	Sweden	Case-Control	1,1-dichloro-2,2-bis (p-chlorophenyl)-ethylene (p,p′-DDE)	Observed an increased risk to develop type 2 diabetes against p,p'-DDE exposure (OR: 5.5 [95% CI 1.2, 25])	(29)
11	Great Lakes region of North America	Cohort	Persistent organic pollutants (POPs) [*p, p*′-diphenyldichloroethene (DDE) and polychlorinated biphenyls (PCBs)]	DDE was found significantly associated with incident diabetes	(31)
12	Taiwan	Cohort	PCBs and polychlorinated dibenzofurans (PCDFs)	The diabetes risk to members of the Yucheng cohort relative to their reference subjects was significantly increased for women (odds ratio [OR] 2.1 [95% CI 1.1–4.5]), In chloracne exposed women diabetes risk is highly significant [AOR of 5.5 (2.3–13.4)]	(32)
13	USA	Case-Control	Persistent organic pollutants (POPs) [hexachlorobenzene (HCB)]	Plasma HCB concentration was positively associated with incident T2D [pooled odds ratio (OR) 3.59 (95% CI: 1.49, 8.64, ptrend = 0.003)	(33)
14	USA	Cohort	Urinary BPA	Positive association between increasing levels of urinary BPA and diabetes mellitus [OR 1.68 (1.22–2.30) (ptrend = 0.002)] was observed	(34)
15	China	Cross-sectional	Urinary BPA	The participants in the highest quartile of BPA had the highest prevalence of generalized obesity [odds ratio (OR) = 1.50; 95% confidence interval (CI) = 1.15–1.97], abdominal obesity (OR = 1.28; 95% CI = 1.03–1.60), and insulin resistance (OR = 1.37; 95% CI = 1.06–1.77)	(36)
16	USA	Nested Case-control	BPA and phthalates	Urinary concentrations of total phthalate metabolites were associated with T2D in the NHSII [OR comparing extreme quartiles = 2.14; 95% CI: 1.19, 3.85; p(trend) = 0.02]. Summed metabolites of butyl phthalates or di-(2-ethylhexyl) phthalates were significantly associated with T2D only in the NHSII; ORs comparing extreme quartiles were 3.16 (95% CI: 1.68, 5.95; ptrend = 0.0002) and 1.91 (95% CI: 1.04, 3.49; ptrend = 0.20), respectively	(37)
17	USA	Nested Case-control	Persistent organic pollutants (POPs)	POPs (in particular, trans-nonachlor and highly chlorinated PCBs) showed non-linear associations with diabetes risk. POPs showed strong associations with type 2 diabetes at relatively low exposure	(38)
18	Germany	Nested case-control	PCBs and pesticides	Increased chance for incident diabetes for PCB-138 and PCB-153 with an odds ratio (OR) of 1.50 (95%CI: 1.07–2.11) and 1.53 (1.15–2.04) was observed	(242)
19	India	Case-Control	Serum BPA	Serum levels of BPA were significantly higher in patients with T2DM compared to control individuals and positively correlated to poor glycemic control and insulin resistance	(243)
**BLOOD PRESSURE/HYPERTENSION**					
20	USA	Cross-sectional analysis	Phthalates	Dietary phthalate exposure is associated with higher systolic BP in children and adolescents	(59)
22	Taiwan	Cross-sectional study	Dioxins	Elevated dioxin levels associated with increases in BP that raises metabolic syndrome risk	(60)
23	Japan	Cross-sectional	Dioxins	Polychlorinated dibenzo-p-dioxins showed small but significant associations with high blood pressure	(244)
24	Japan	Cross-sectional study	Dioxins	High dioxin levels associated with an increases in BP	(245)
25	USA	Cross-sectional study	Dioxins	High dioxin levels associated with increases BP in women	(246)
26	USA	Cross-sectional study	Dioxins	High dioxin levels associated with increases in BP	(247)
27	Greenland	Cross-sectional study	PCBs	High PCB levels associated with fish consumption associated with increased BP levels	(248)
28	Iceland	Cross-sectional analysis	PCBs	High PCB levels associated with high BP	(249)
29	Sweden	Cross-sectional analysis	PCBs	High PCB exposure associated with an increased risk of incident hypertension	(246)
30	USA	Cross-sectional analysis	PCBs	High dioxin levels associated with increases in BP	(247)
31	Korea	Cross-sectional analysis	BPA	Highest quartile BPA excretion associated with 1.27-fold increased risk of hypertension	(48)
32	Iran	Case-control study	BPA	Elevated Urinary BPA associated with increased risk of hypertension	(250)
**RENAL FUNCTION**					
33	Taiwan	Cross-sectional study	Dioxin	Highest quartile exposure associated with a 15–22 ml/min/1.73 m^2^ reduction in eGFR; Men with dioxin levels greater than reference group levels had a 2.2-fold increased risk of hyperuricemia	(251)
34	USA	Cross-sectional study	Mixture of POPs	Exposure leads to risk of hyperuricemia	(252)
35	USA	Cross-sectional study	Perfluoroalkyl chemicals (PFCs)	Interquartile rise in PFOA excretion associated with an 0.75 ml/min/1.73 m^2^ decline in eGFR	(88)
36	USA	Cross-sectional study	PFC	Highest quartile of PFOA and PFOS associated with a 1.8-fold increased risk of CKD; Highest quartile of PFOA associated with a 1.97-fold increased risk of hyperuricemia	(34)
37	USA	Cross-sectional study	PFC	Highest quartile of PFOA associated with 1.62-fold increased risk of hyperuricemia	(253)
38	Japan	Data mining analysis	PCBs	Direct correlation between serum PCB and uric acid concentrations	(254)
**WOMEN REPRODUCTIVE HEALTH**					
**Endometrosis**					
39	Italy	Case control study	BPA/BPB	Findings strongly suggest the existence of a relationship between occurrence of endometriosis and the presence of BPA and/or BPB in the serum	(100)
40	Korea	Case-control study	Phthalate	Study has shown that the plasma levels of monoethylhexyl phthalate, as well as DEHP, are significantly higher in those with advanced-stage endometriosis	(101)
41	France	Case-control study	PCBs, BFRs and OCPs	Severe cases of endometriosis (Stages IIIIV), shows that internal exposure levels of several dioxins, PCBs, BFRs and OCPs in adipose tissue are higher in individuals presenting deep infiltrating endometriosis (DIE) compared to controls	(104)
42	Spain	Case-control study	Dioxins and PCBs	Dioxins and PCBs in adipose tissue were significantly higher in patients with DIE in comparison with the control group (*p* < 0.05)	(255)
43	USA	Case-control study	Organochlorine pesticides (β-hexachlorocyclohexane)	Serum concentrations of β-HCH and mirex were positively associated with endometriosis	(105)
44	USA	Case-control study	Phthalates	Study suggest that phthalates may alter risk of a hormonally-mediated disease among reproductive-age women	(107)
**Puberty**					
45	Thailand	Cross-sectional study	Phthalates	Precocious puberty girls had an association with increased mono-ethyl phthalate (MEP) concentration	(74)
46	USA	Longitudinal cohort study	Phthalates	Findings suggest that female reproductive development may be more vulnerable to the effects of phthalate or BPA exposure during specific critical periods of *in utero* development.	(88)
**POLYCYSTIC OVARY SYNDROME**					
47	China	Case–control study	BPA	Results suggest that in women with PCOS, BPA may affect ovarian follicles and, therefore, reduce ovarian reserve	(91)
48	United Kingdom	Case-control study	BPA	Higher BPA levels in PCOS women compared to controls and a statistically significant positive association between androgens and BPA point to a potential role of this endocrine disruptor in PCOS pathophysiology	(93)
**OBSTETRIC OUTCOMES**					
49	China	Case-control study	BPA	*In utero* exposure to BPA during pregnancy may be associated with decreased birth weight in offspring	(109)
50	Taiwan	Cohort Study	BPA	Elevated prenatal BPA exposure increased the risk of lower birth weight, smaller size for gestational age, and adverse actions of adipokines in neonates, especially in male infants	(110)
51	China	Nested Case-Control Study	BPA	Prenatal exposure to higher levels of BPA may potentially increase the risk of delivering LBW infants, especially for female infants	(111)
52	China,	Longitudinal birth cohort	BPA	Maternal exposure to low-level BPA may affect birth length among male neonates	(113)
53	Multi country	Case-control study reproductive aged couple	BPA	Preconception maternal and paternal urinary concentration of BPA and specific phthalate metabolites may be associated with smaller birth size and increased gestational age	(115)
54	China	Longitudinal Healthy Baby Cohort	Methyl paraben (MeP)	Maternal urinary levels of MeP were positively associated with length at birth in boys	(116)
55	USA	Prospective birth cohort study	Organochlorine pesticides (OPPs), perfluoroalkyl substances (PFAS), polybrominateddiphenyl ethers (PBDEs)	Gestational OPP, Pb, and PFAS exposures were most strongly associated with lower birth weight	(117)
**MALE REPRODUCTIVE DISORDER**					
**Male puberty**					
56	USA	Cohort	BPA	Observed relationships between BPA levels and increased sex hormone binding globulin (SHBG)as well as decreased Total and Free Testosterone	(121)
57	Taiwan	Cross-sectional study	Phthalic acid esters (PAEs)	Study suggests that PAE (specifically, DEP, DnBP, DiBP, and DEHP) exposure is associated with abdominal obesity in adolescents and that the APs for abdominal obesity are more sensitive than BMI for measuring obesity among adolescents	(122)
58	China	Cross-sectional study	BPA	Findings indicate an association between peripubertal BPA exposure and earlier pubertal onset, but delayed pubertal progression, in boys	(123)
59	Spain	Cross-sectional study	Organophosphate (OP)	Exposure to OP pesticides may be associated with decreased sperm counts and motility and altered reproductive hormone levels	(125)
60	China	Cohort study	BPA	Exposure to BPA in the workplace could have an adverse effect on male sexual dysfunction	(256)
61	South Africa	Cross-sectional study	DDT	Study found evidence that indicated that non-occupational exposure to p,p9-DDT and its metabolite p,p9-DDE has an effect on seminal parameters	(126)
62	USA,	Cross-sectional	BPA	Human exposure to BPA may be associated with reduced semen quality and increased sperm DNA damage	(128)
63	Russia	Longitudinal Study	Dioxin	Higher peripubertal serum Dioxin were associated with poorer semen parameters	(131)
**Cryptorchidism**					
64	Canada	Case-Control Study	PBDEs	Maternal exposure to PBDEs may be associated with abnormal migration of testes in the male fetus	(133)
65	Finland/Denmark	Case-Control Study	PCBs, polychlorinated dibenzo-p-dioxins and furans (PCDD/Fs)	Prenatal exposure to PCDD/Fs and PCDD/F-like PCBs may be associated with increased risk for cryptorchidism	(134)
66	Spain	Case-control study nested in a birth cohort	BPA, benzophenones (BPs) and parabens (PB)	Statistically significant association was observed between exposure to BPA and propyl-PB and the risk of malformations [adjusted odd ratio (95% CIs) in the third tertile of exposure: 7.2 (1.5–35.5) and 6.4 (1.2–35.5) for BPA and propyl-PB, respectively	(135)
**NEURODEVELOPMENTAL DISORDER**					
68	Canada	Prospective cohort	BPA	Maternal urinary BPA concentration during pregnancy was associated with some aspects of children's behaviors at 3 years of age	(145)
69	Korea	Prospective cohort study	BPA	Prenatal and postnatal BPA exposure is associated with social impairment at 4 years of age, particularly in girls	(146)
70	USA	Birth cohort	BPA	Gestational BPA exposure affected behavioral and emotional regulation domains at 3 years of age, especially among girls	(147)
71	USA	Cohort	BPA	Prenatal exposure to BPA may affect child behavior, and differently among boys and girls	(148)
72	USA	Cohort	BPA	Positive associations between prenatal BPA and symptoms of depression and anxiety were observed among boys.	(149)
73	USA	Prospective multiethnic cohort	Phthalate	Children clinically diagnosed with conduct or attention deficit hyperactivity disorders.	(152)
74	Korea	Cohort Study	PCBs and DEHP	PCBs and DEHP was associated with adverse neurodevelopmental performances among the children aged 1–2 years	(153)
75	USA	Birth cohort study	Organophosphate (OP) pesticides	OP pesticides was associated with poorer intellectual development in 7 year-old children from an agricultural community	(154)
76	USA	Prospective birth cohort,	PBDEs	Prenatal exposure to PBDEs was associated with lower IQ and higher hyperactivity scores in children	(155)
77	USA	Cross-sectional study	PBDEs	Study demonstrates neurodevelopmental effects in relation to cord blood PBDE concentrations	(156)
78	Canada	cohort	PCBs	Higher prenatal PCB exposure was associated with decreased Fagan Test of Infant Intelligence (FTII) novelty preference, indicating impaired visual recognition memory	(158)
79	USA	Cross-sectional study	PCBs	Exposure to PCBs may be associated with some measures of memory and learning and depression among adults 55–74 years of age	(159)
80	USA	Cross-sectional study	PBDEs	PBDEs and PCBs may interact to affect verbal memory and learning among persons 55–74 years old	(160)
**THYROID HOMEOSTASIS**					
81	USA	Observational and cross-sectional study	Phthalate	Found relationship between urinary phthalate metabolites and serum thyroid hormone levels	(166)
82	USA	Cohort	phthalate	Increased maternal urinary concentrations of monoethyl phthalate (MEP) are associated with decreases in maternal TT4	(167)
83	Japan	Prospective birth cohort	BPA	Positive association between cord blood BPA and T, E2, and P4 among boys	(58)
84	USA	Prospective pregnancy and birth cohort	BPA	Prenatal BPA exposure may reduce TSH among newborn girls, particularly when exposure occurs later in gestation	(169)
85	USA	Pilot case-control study	BPA	Found associations of urinary BPA with TSH	(170)
87	Canada	Prospective cohort	PBDEs and PCBs	Exposure to PBDEs and PCBs in pregnancy may interfere with thyroid hormone levels	(171)
88	Multi country	Cohort	PCBs and p,p'-dichlorodiphenyldichloroethene (p,p'-DDE)	Early life exposure to PCB and p,p'-DDE was associated with newborn TSH levels	(173)
89	USA	Prospective pregnancy and birth cohort	PBDEs	Maternal PBDE exposure, are associated with maternal concentrations of T4 and T3 during pregnancy	(174)
90	USA	Observational prospective cohort study	PBDEs	PBDEs may be affecting thyroid regulation throughout pregnancy	(175)
91	Korea	Cohort	PCBs and PBDEs	PCBs and PBDEs exposure among pregnant women are clearly related with potential for disrupting thyroid hormone homeostasis in the present study	(176)
**CANCER**					
92	USA	Cross-sectional study	PCB	Suggest a link between environmental exposures to PCB and breast cancer	(192)
93	USA	Prospective cohort study	Organophosphate and organochlorine	Increases the risk of prostate cancer	(198)
94	USA	Case-control study	Phthalate	Exposure to diethyl phthalate, the parent compound of MEP, may be associated with increased risk of breast cancer	(257)
95	Taiwan	Nested case-control study	Phthalate	DEHP, BBzP, and DiBP exposure were associated with prostate cancer occurrence in abdominally obese men	(258)

The breadth of this review identifying negative human health impacts of various EDC's strongly suggests that there are significant risks to human health and a precautionary approach and human bio-monitoring of EDC's is warranted.

## Effects of EDCs on Glucose Metabolism and Obesity

**Figure 3:**
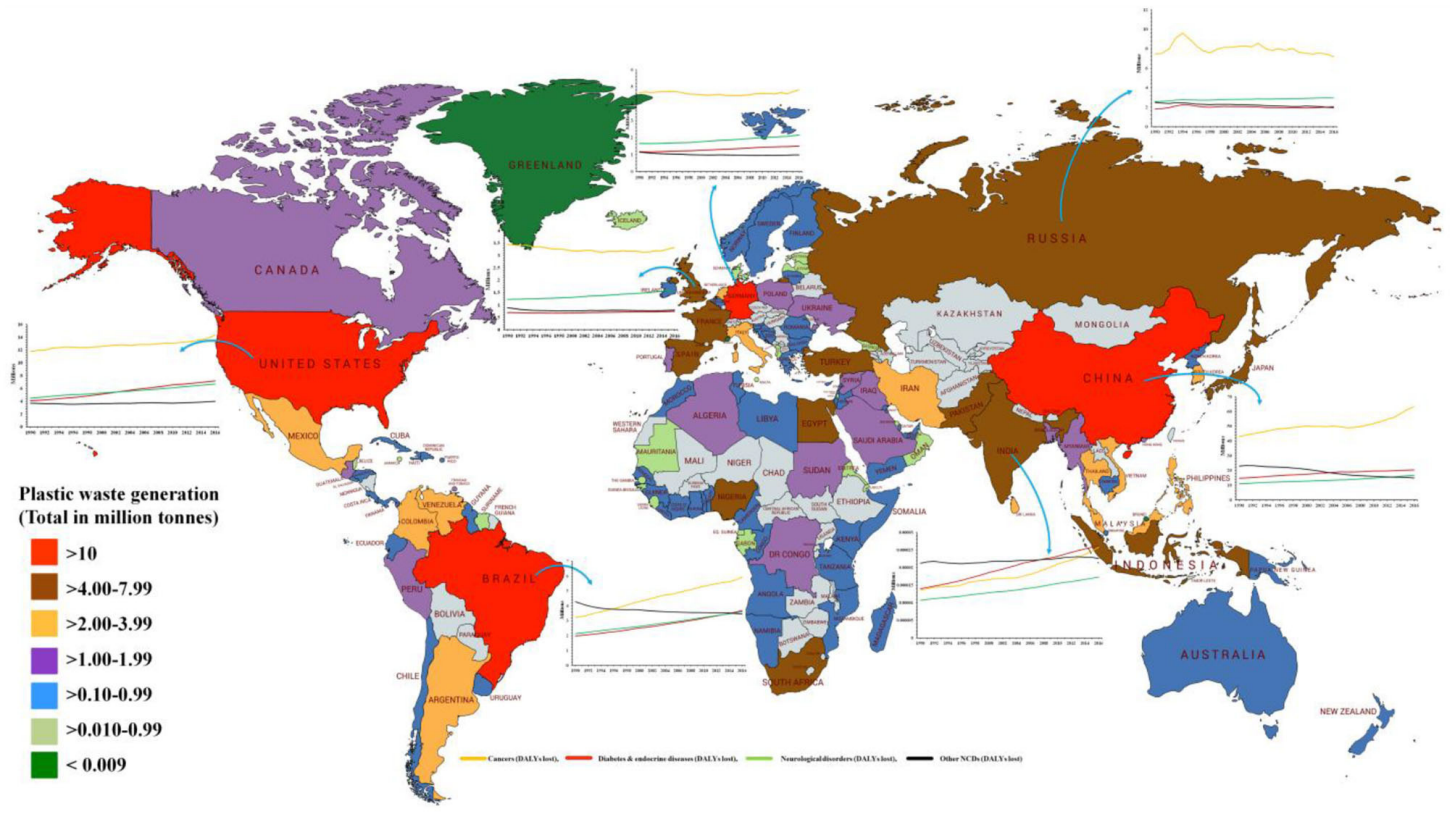
Total plastic waste generation by country, measured in million tons per year for the year 2010 and disease burden from non-communicable diseases (from 1990 to 2016). Total disease burden from non-communicable diseases (NCDs), measured in DALYs (Disability-Adjusted Life Years) per year. DALYs are used to measure total burden of disease—both from years of life lost and years lived with a disability. One DALY equals 1 lost year of healthy life. Base map courtesy of mapchart.net (http://www.mapchart.net) [Source: (10, 11)].

**Figure 4:**
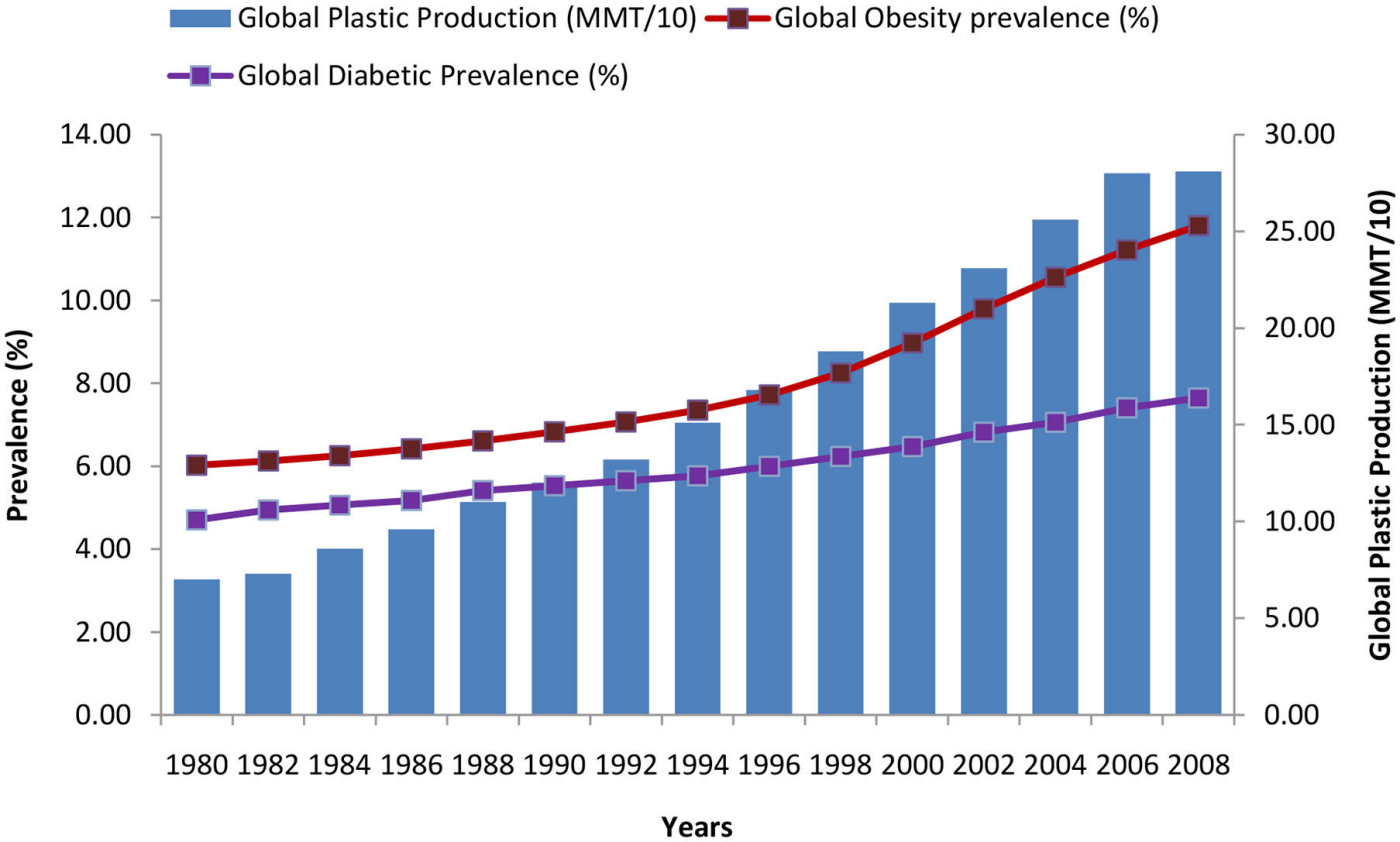
Trends of global diabetes, obesity, and plastic production during the period of 1980–2008 [Data source: https://ourworldindata.org/plastic-pollution and (9, 12)].

Over the past several decades, obesity and type two diabetes rates have increased markedly in all age groups worldwide and particularly in developed countries. Obesity is frequently associated with other morbidities such as metabolic disorders (including metabolic syndrome, type 2 diabetes, cardiovascular diseases, pulmonary complications, and liver disease), psychological/social problems, reproductive abnormalities and some forms of cancers. An amalgamation of lifestyle, genetic and environmental factors probably accounts for the quick and considerable increase in obesity rates. An increase in the occurrence of metabolic diseases has been noted in last several decades, which coincide, with the significant changes in the chemical environment around us, which came in practice during the same period. Velmurugan et al. (9) has demonstrated that the common risk factors for diabetes may not be the sole contributor, while other factors such as production of EDCs (plastics, plasticizers, pesticides, e-waste, food additives) has been found to be positively associated with the growing trend of the global diabetes incidence in last few decades (Figures 3, 4).

This change in our chemical environment seems to be a contributing factor in this “obesity pandemic” apart from genetic factors and modern lifestyle that embraces high energy intake, reduced physical activity, sleep deprivation and near stable home temperatures. This has led to the hypothesis that some of these environmental chemicals disrupt the endocrine process and lead to obesity by interfering with various endocrine aspects of metabolism (13, 14). This hypothesis garners support from animal as well as epidemiological studies, which have revealed that a diverse array of environmental chemicals can influence adipogenesis and obesity; however, its extent, exact mechanism and epidemiology are yet to be elucidated. The EDCs linked with obesity and adiposity include several POPs (15–18), BPA (19, 20), and phthalates (21–23). It has been suggested that *in utero* or childhood exposure to certain EDCs predisposes some individuals to obesity, highlighting the importance of timing of exposure (17, 21, 22, 24, 25). Such EDCs have been referred to as “environmental obesogens” (26). The term “metabolic disruptors” has also been suggested as many of these chemicals be associated with other metabolic diseases such as metabolic syndrome, insulin resistance and type 2 diabetes as well (17, 27). Several studies have found association of type 2 diabetes and/or its risk factors with PBCs, DDE, organochlorine pesticides, HCB, dioxins, BPA and phthalates (28–37). Some prospective studies have interestingly concluded that no single POP could significantly predict T2D, but some combination of POPs could predict the future risk of T2D (28, 31–33, 38). This also lends support to the concern that exposure to EDCs as mixtures that occur in the environment may have greater significance than that to single chemicals tested in laboratories. In addition, several studies have shown association of gestational diabetes to phthalates, BPA and PCB (39–42). Increased risk of non-alcoholic fatty liver disease has been linked to BPA (43) as well as cardiovascular risk factors are found to be linked to both organochlorine pesticides (44–46) and BPA (47, 48).

EDCs alter metabolic balance through multiple mechanisms including alterations in peroxisome proliferator-modulated pathways (49), adipogenesis (50), pancreatic β-cell function (51–53) and hypothalamic neuropeptides (54, 55). In addition, decreased physical activity in relation to whole-body energy balance has been reported in mice that were exposed to BPA either during a perinatal period or in adult life (56, 57). However, most of the evidence regarding the mechanism of action of EDCs comes from experimental studies. In a recent study, it was shown that prenatal exposure to BPA and certain phthalates might affect the levels of adiponectin and leptin in human fetus (58). A novel hypothesis advanced is the role of gut microbiota in EDC-induced type 2 diabetes. It is thought that EDCs not only cause dysbiosis of gut microbiota but also undergo metabolism by gut microbes, and both these processes alter the gut neuroendocrine regulation of metabolism leading to insulin resistance and type 2 diabetes (9).

## Effect of EDC on Blood Pressure

To elucidate associations of urinary EDC's like phthalate with blood pressure (BP), a cross-sectional analysis of 6–19 years children in the 2003–2008 National Health and Nutrition Examination Survey (NHANES) was carried out which reveals an increase in blood pressure associated with 3-fold increase in urine phthalate (DEHP) (59). A study conducted in people living near a dioxin-contaminated area showed that increasing serum dioxin levels are correlated with elevated diastolic blood pressure. In addition, the prevalence of hypertension was also found to be correlated with serum PCDD and PCDF levels in Taiwanese and Florida adults with dioxin exposure (60, 61). Similarly, high serum PCB Level was also found to be associated with elevated blood pressure in NHANES 1999–2002 and NHANES 1999–2004 participants (62). Studies derive conclusions about the effects of EDCs on hypertension but further studies with larger diverse cohorts are now needed to verify an association between EDC's exposure and risk of hypertension.

## EDCs: Female and Male Reproductive Health

### Female Reproductive Disorders: Role of Environmental EDCs

Several EDCs are known to exert effects on female reproductive hormones and their receptors through oestrogenic, anti-estrogenic, androgenic and anti-androgenic mechanisms. Some of these EDCs include BPA and phthalate, which are classified as xeno-oestrogens. They act by both receptor-dependent and receptor-independent mechanisms, either directly by binding to estrogen receptors, increasing aromatase activity and increasing estrogen sensitivity, or indirectly by their effect on gonadotropin-releasing hormone, leading to an increase in endogenous estrogen production (63–65). EDCs are thought to affect the menstrual cycle, alter fertility and oogenesis and are implicated in diseases such as polycystic ovary syndrome (PCOS) and endometriosis. Hauser et al. (66) documented the first-ever adverse effect of urinary phthalate metabolites by using *in vitro* fertilization (IVF) where they found that concentrations of urinary metabolites of di(2-ethylhexyl) phthalate (DEHP) and di-isodecyl phthalate (DiDP) was inversely related with oocyte yield and number of metaphase II oocytes at retrieval, whereas only metabolites of di-isononyl phthalate (DiNP) and di-isodecyl phthalate (DiDP) was linked with reduced fertilization rate. However, the exact mechanism of action of these chemicals is not yet clearly understood, but it is likely that multiple pathways are involved including hormone receptors.

### Female Puberty

Puberty is a phase of transition between adolescence and adulthood during which the reproductive system reaches full maturity (67). The onset of puberty involves a complex neuroendocrine mechanism and influenced by several factors including ethnicity, genetics, nutrition and environmental factors (68, 69). Recently early onset of puberty in girls has been noted across several countries and a role of environmental pollutants has been implicated, notably that of BPA, phthalates, and organohalogens (70). Puberty is brought about by activation of the hypothalamic-pituitary gonadotropic cells-gonadal axis. The activation of this axis is thought to be regulated by a novel neuropeptide and its receptor, which are in turn modulated through sex steroid receptors in hypothalamic neurons (71). It is possible that oestrogenic EDCs or xeno-oestrogens act through the sex steroid receptors to bring about early puberty. During childhood, the levels of sex hormones in blood are very low. However, it has been found that estrogen and androgen receptors are expressed since a very early age (72). Therefore, even a small change in these levels through an exogenous source may bring about several pubertal changes (73). In several studies, early onset of pubertal changes in girls has been reported to be associated with phthalates (74–77), BPA (78, 79), and organohalogens (80–82), whereas in other studies, no such association (30, 83, 84) or even inverse association has been reported (85, 86). Another study found a non-linear association of BPA and phthalate metabolites with pubertal development (87). However, it is possible that these differences result from variation in environmental exposure, timing of exposure and non-linear dose-response patterns of EDC effects. Exposure to EDCs during prenatal and perinatal period has also been thought to be linked with early onset of puberty in girls as well (88).

### Polycystic Ovary Syndrome (PCOS)

Most of the information on direct effects of EDCs on fertility is available from *in vitro* and animal studies. A few human studies have been carried out in women attending fertility clinics and have provided variable results. Some of these studies have shown an association of EDCs directly with infertility and its parameters, whereas others have correlated them with PCOS, which is a complex endocrinopathy leading to infertility and insulin resistance. A negative association between urinary concentrations of phthalate metabolites and serum inhibin B levels was found, suggesting a detrimental effect of phthalates on growing antral follicles (89). In the Longitudinal Investigation of Fertility and the Environment Study, concentrations of phthalates, but not BPA in women were associated with longer time to pregnancy (90). In other studies, BPA was found to be associated with reduced ovarian reserve (91), lower antral follicle count (92) and PCOS (93, 94) in infertile women. Higher quartiles of urinary BPA concentration were also shown to be associated with increased odds of implantation failure (95). One of the studies showed that high PCB and pesticide concentrations in the follicular fluid correlated with poor embryological intracytoplasmic sperm injection outcomes (96). Another study documented higher serum concentrations of perfluorinated compounds and phthalate metabolites was present in women with PCOS (97). DDT was found to be associated with altered hormonal levels in Chinese women with PCOS suggesting a role in its pathogenesis (98). In a large American cross-sectional study involving more than 30,000 women, researchers found an association between EDCs and earlier age at menopause, and they also identified 15 EDCs (including 9 PCBs, 3 pesticides, 1 furan and 2 phthalates) that needed further evaluation for potential adverse effects on ovarian function (99).

### Endometriosis

Endometriosis is the presence of ectopic endometrial tissue, which is a cause of pain and infertility. Relatively few studies have explored the role of EDCs in endometriosis. However, its association has been reported with higher blood or urinary levels of BPA (94, 100), various phthalates (101–103) and organochlorine pesticides (104, 105). On the other hand, no association or inverse association with phthalates was observed in studies on infertile Japanese women and American women, respectively (106, 107). A recent meta-analysis of past epidemiological studies has recorded a significant constant increase in the risk of endometriosis to polychlorinated bisphenyls (PCBs) group (OR = 1.58; 95% CI: 1.18–2.12), organochlorine pesticides (OCPs) group (OR = 1.40; 95% CI: 1.02–1.92) and phthalate esters (PAEs) group (OR = 1.27; 95% CI: 1.00–1.60), while BPA showed no significant association with endometriosis (108).

### Obstetric Outcomes

*In utero* exposure to EDCs through trans-placental route has become a concern as it is thought that small endocrine imbalance during fetal development can lead to significant and lasting changes. Several researchers investigated this in the last decade. *In utero* BPA exposure was found to be associated with decreased birth weight (109–112) and increase in gestational length (112, 113). However, Shoaff et al. (114), did not find any association between prenatal phthalate exposure and birth size or duration of gestation. In another study, both maternal and paternal BPA and phthalate concentration were found to be associated with smaller birth size and increased gestational age (115). Though both maternal and paternal exposure to these chemicals will be similar as they are exposed to similar environment, paternal EDC levels likely reflect maternal exposure and do not directly affect the fetal exposure. Maternal urinary levels of paraben were found to be associated with increased length of male neonates in a Chinese cohort (116). An American study on several classes of EDCs showed that gestational exposure to perfluoroalkyl substances and organophosphate pesticides was associated with reduced birth weight while phthalates, BPA, PCBs, PBDEs (Polybrominated diphenyl ethers) or OCPs (organochlorine pesticides) had either small or no association with differences in birth weight (117).

### Male Reproductive Disorders: Role of Environmental EDCs

EDCs are thought to affect the male reproductive system as well. Human studies indicate that these chemicals affect pubertal growth and semen quality and cause defects such as hypospadias and cryptorchidism. Well over two decades back, Sharpe and Skakkebaek (1993) proposed that several male reproductive disorders such as low sperm counts, cryptorchidism and hypospadias may share a common fetal origin and that environmental exposure to oestrogenic chemicals might play a key role (118). Though this estrogen hypothesis was later refuted, except diethylstilbestrol, it propelled scientific research on effects of EDCs on male reproductive system. It is now thought that EDCs act on male reproductive system by disrupting fetal endocrine balance through their action on steroid hormone receptors (SHRs) or interference with the synthesis, kinetics or metabolism natural hormones (World Health Organization [WHO] and United Nations Environment Program 2012). Sperm function affected by reactive oxygen species generated during metabolism of these chemicals is another possible effect of EDCs leading to infertility (119).

### Male Puberty

The effect of EDCs on male puberty has received relatively less attention compared with that on female puberty, and the results of these studies show wide discrepancies. These studies unlike those on semen quality mostly show correlations with rapidly metabolizing chemicals. Association of early pubarche in boys was noted with Polybrominated diphenyl ether (PBDE) (86) and some phthalates (120), while inconsistent or no association of phthalates and BPA with puberty in boys was found in other studies (87, 121). In another study, monomethyl phthalate exposure was found to be inversely associated with pubarche among boys while there was no association with other phthalate esters (122). Wang et al. (123) documented that peripubertal BPA exposure in boys was associated with earlier pubertal onset, but delayed pubertal progression.

### Semen Quality

There are numerous causes of infertility in men. Even though sperm count does not associate specifically with fertility, it is documented that men with very low sperm counts frequently have fertility problems. In 1996, in their review article, Toppari et al. expressed concern regarding decreasing semen quality in the last few decades, and they advanced the hypothesis that exposure to environmental chemicals may be responsible for adverse trends in male reproductive health (124). Several EDCs have been found to affect semen parameters adversely. These include organophosphate pesticides (125–127), BPA (128), perfluorinated compounds (129), phthalates (130), and organochlorines (131). It was observed that adult life exposure to PBC affects mainly sperm motility while perfluorinated compounds alter sperm morphology primarily. However, the overall impact of EDCs on male fertility is not clear so far. Though sperm count and morphology appear to be altered to some extent, the effect on fertility seems to be minor at best.

### Cryptorchidism

Cryptorchidism is the condition in which the testis does not descend completely to the bottom of the scrotum. Its incidence has been documented to increase from 1–3% in the late 1950s to 7–9% in the 2000s (132). This condition shows familial clustering suggesting a role of genetic and environmental factors. Known risk factors for cryptorchidism include maternal gestational diabetes and maternal use of alcohol, acetaminophen or nicotine-containing substances. In recent research, cryptorchidism has been found to be associated with prenatal or early childhood exposure of several chemicals including PBDE (133, 134), BPA (135), parabens (135), and PBCs (134). Other researchers have found no association of cryptorchidism with PBBs (136), organochlorine pesticides (137), perfluorinated compounds (138, 139), or PCBs (140). Because several environmental factors are also associated with cryptorchidism, the effects of individual factors are difficult to establish and the possibility of confounders cannot always be eliminated in these studies.

### Hypospadias

Hypospadias is a congenital malformation in which the urethral folds do not fuse properly. A meta-analysis suggested an association between pesticide exposure in men and risk of hypospadias in their offspring (141). Another study suggests that exposure to HCB and p,p′-DDE during fetal life may be a risk factor for hypospadias (142). However, no significant association of exposure to PCBs (140, 143), organochlorine pesticides (137), PBBs (136), PBDE (143), or perfluorinated compounds (144) with hypospadias was noted in other studies. Hypospadias is a relatively uncommon condition; therefore, most of these studies face the problem of small sample size and are not statistically robust.

Overall, the effect of individual EDCs on reproductive system appears uncertain; however, it is possible that some additive or synergistic effects of mixtures do exist. Conflicting results and differences in study variables make it difficult to establish the role and extent of effects of EDCs either individually or in combinations. Large-scale studies taking into account the exposure of all known EDCs in a uniform manner will be needed to explore such complex association.

## Role of EDCs in Neurodevelopmental Disorders

Prenatal exposure to EDCs is thought to affect brain development in the fetus, and some neurodevelopmental abnormalities have been attributed to them. Prenatal BPA exposure has been linked to poor social behavior (145, 146), higher anxiety and depressive behaviors (147). Some studies have suggested a sex-specific effect of BPA on behavior. A study showed that poor social behavior was found to be more pronounced in girls (146), while a study showed that significant positive associations were found between prenatal BPA and emotionally reactive and aggressive behavior in boys (148). In another study, prenatal BPA exposure was noted to be associated with increased anxiety and depression in boys but not in girls (149). The gender difference is likely due to the close relation of brain development with gonadal hormones as proven by animal studies. The brain, particularly hypothalamus, has been shown to be affected by oestrogenic EDCs in a sex-, time- and exposure-dependent manner (150). *In utero* exposure to phthalates is shown to be associated with adverse cognitive and behavioral outcomes such as lower IQ, attention deficit, hyperactivity and poorer social communication in children (151–153). Other EDCs such as organophosphates (152, 154), PBDEs (155, 156), perfluorinated compounds (157), and PCBs (153, 158) have also been linked to adverse neurodevelopmental performance, behavioral problems and lower IQ to varying degrees. There is also some evidence that some PBDEs and PCBs affect neuropsychological status in adults (159, 160). Animal studies have provided ample evidence that EDCs act on developing brain through multiple mechanisms: (a) steroid hormones and receptors, (b) neuroendocrine system, and (c) epigenetic changes (161). One of the main biological mechanisms proposed for adverse neurodevelopmental effects of prenatal EDC exposure is disturbance of thyroid hormone homeostasis, which is critical for fetal brain development (162).

## Thyroid Homeostasis and EDCs

Thyroid hormones are controlled through the hypothalamic–pituitary–thyroid axis, and they are vital for the regulation of several biological systems and can be disrupted by EDC exposure. Specifically, the phenols and phthalates are thought to act directly on the thyroid hormone receptor, and studies have shown that these compounds are thyroid receptor antagonists. Epidemiological studies have shown that cognitive functions of the offspring may be adversely affected by even a marginal lowering in thyroxin levels in a pregnant woman (163–165). Thus, exposure to thyroid-disrupting chemicals may have significant consequences for public health even if they bring about a small reduction in thyroid hormone levels. Several studies have evaluated the EDC exposure effect on thyroid hormones in humans. Most of these have explored the effect on pregnant women and new-borns. A study reported that urinary concentrations of phthalate metabolites were seen to be associated with lower T4 and T3 or higher thyroid-stimulating hormone (TSH) levels in adults (166). In another study, prenatal exposure to phthalates was found to be inversely associated with both total serum thyroxine in pregnant women and newborns as well as TSH in newborns (167). The association with BPA is more variable in several studies. Two studies found no association between thyroid hormone levels and maternal BPA exposure (168) and cord blood BPA levels (58), respectively. Another study reported no association between maternal urinary BPA concentrations and thyroid hormones measured in cord serum when analyzed for all newborns, but they found that in female newborns, lower cord TSH was associated with the 10-fold increased BPA in maternal urine (169). In the analysis of data from the U.S. National Health and Examination Survey, urinary BPA showed a negative relationship with serum TSH levels in adults (166), while another European study concluded that a significant positive association was observed between urinary BPA and serum TSH in adult women (170). A study found that there was a negative correlation between PCB levels and maternal free T3 but not with cord blood (171). However, a systematic analysis did not find any correlation between PCBs and thyroid hormones in either pregnant women or newborns (172). In a study including three European cohorts, the researchers concluded that PCB was associated with lower TSH levels in newborns (173). In another study, in the case of PBDEs, negative correlation was found with maternal and cord blood thyroid hormones (171), while positive correlation was seen with maternal blood thyroid hormone levels in two other studies (174, 175). Organochlorine pesticides were noted to be associated with reduction in thyroid hormones (176).

## Renal Function and EDCs

Chronic kidney disease (CKD) is a worldwide health menace of growing concern, and its prevalence all over world is estimated at 13.4% (177). Kidneys serve as the filtration unit and thus are exposed to all compounds in a greater degree compared to other organs. Humans are not exposed to a single compound but to mixture of compounds and they are likely to affect the kidneys as well. Without a doubt, many non-persistent EDCs, e.g., phthalates, bisphenols, benzophenones, and parabens, share common exposure sources such as plasticware and personal care products in various things.

There are growing evidences suggesting that non-persistent endocrine disrupting chemicals (EDCs), are linked with adverse kidney function (178). Studies also showed that exposure to di-(2-ethylhexyl) phthalate (DEHP) through the consumption of contaminated milk was associated with micro-albuminuria in children (179, 180). The US National Health and Nutrition Examination Survey (NHANES) delineated the association of urinary phthalate metabolites with CKD markers such as albumin-to-creatinine ratio (ACR), estimated glomerular filtration rate (eGFR), and urinary protein-to-creatinine ratio (UPCR) has also been revealed in a population of children (181, 182). Urinary BPA levels were also associated with ACR among the US children (183), as well as Chinese adults (184). Patients on dialysis have more exposure to BPA due to daily use of dialysis tubing. BPA levels are higher in patients undergoing hemodialysis and peritoneal dialysis, than in healthy controls (185). NHANES 2003–2006 Estimated Glomerular Filtration Rate (eGFR) among a general population of the US adult females shows positive association with urinary BPA levels but not in the adult males (186).

## EDCs and Hormone-Dependent Cancers

A large fraction of EDCs acts through oestrogenic and androgenic receptors; therefore, researches have focused on their relation to hormone-dependent cancers. Such neoplasm's include breast, endometrial, ovarian, and cervical cancers in women and prostate cancer in men. The incidence of these cancers has risen at a higher rate over the last few decades (187–189). As other non-communicable disease, this increase is multifactorial, and change in lifestyle, industrialization and EDCs are thought to be responsible at least in part (161).

The development and progression of estrogen-dependent malignancies, particularly breast cancer, depend on prolonged or exaggerated estrogen exposure (190). Approximately 70% of breast cancers are sensitive to estrogen as they show ER-positivity (191). Unopposed estrogen or estrogen-mimicking EDCs can bind to ER, switching on the downstream signaling of estrogen-responsive genes involved in the cell cycle (64, 192, 193). Several oestrogenic EDCs, such as BPA and phthalates, have been shown to induce epigenetic modification or genotoxic effects (27, 194, 195), which can alter gene products and genetics, eventually predisposing to certain diseases. However, only a few human studies have explored this association so far. Although meta-analysis did not find any association between organochlorine pesticides and breast cancer (196), another study linked it to exposure before 14 years of age (197). A study in Mexico showed a positive correlation between MEP, a phthalate metabolite, and breast cancer while other phthalates (MBP, MEOHP, and mono [3-carboxypropyl]) had negative correlation with breast cancer risk. The evidence of any relation between BPA exposure and breast cancer is weak, and it is observed only in animal studies. This may be due to its short half-life so that measurement of exposure is difficult (161). Agricultural Health Study, USA, has found that several pesticides are linked with increased risk of prostate cancer (198, 199). The link between EDCs and other hormone-dependent cancers is tenuous as it is based on a handful of animal and laboratory studies. It has not yet been explored through human studies, and it constitutes a lacuna in our knowledge.

## Endocrine Disrupters Accelerate Aging

Growth is the consequence of a multifaceted interaction of genetic, constitutional, nutritional, endocrine and socio-economic factors as well as psychosocial well-being (200, 201). The evidence is seen in the fact that over the last century, children have gradually become taller and are reaching puberty earlier. For example, the age of menarche in European girls in the middle of the nineteenth century was 16–17 years, in contrast to the current age of 13 years or less. Rampant use of sex steroids as growth promoters has occurred in animal farming, which continues in several countries of the world.

The timing and evolution of reproductive aging depend mainly on genetic and environmental factors. So far, most research has focused on genetic predispositions (202). Nevertheless, the environment probably plays a role, and there is an assumption that EDCs may accelerate reproductive aging, resulting in shortened reproductive lifespan. Recent studies show that exposures to EDC during critical developmental periods, particularly during fetal life and early childhood can cause molecular/cellular changes that modify the function of the affected tissues later in life, a concept referred to as the fetal/developmental basis of adult disease (203). These effects are thought to be brought about by epigenetic modifications. Animal studies show that reproductive aging is accelerated by EDC (MXC) (204), BPA (205), and dioxins (206). Recent epidemiological evidence also links EDC exposures during periods of growth to accelerated menopause [diethylstilbestrol (207) and perfluorocarbons (208)]. BPA has also been shown to promote oxidative stress and inflammation, and it has been suggested that it makes postmenopausal women suffer health effects related to aging (209).

## Dose-Response Dynamics of EDCs

Several biological effects of EDCs are mediated through SHRs (210). Hormone receptors are high-affinity receptors, that is, a small concentration of hormones or hormone mimickers can produce a significant biological effect. In addition, the dose-response curves of these receptors are non-linear; they can be sigmoidal, or more complex, including being non-monotonic. The non-monotonic dose-response (NMDR) curves are often “U-shaped” (with maximal responses observed at low and high doses) or “inverted U-shaped” (with maximal responses observed at intermediate doses) (161). EDCs mimic hormones in their actions including low-dose effects and dose-response patterns, which depend on characteristics of their binding receptors and ligands (161). Due to this property, environmentally relevant concentrations of these chemicals need to be studied thoroughly for their effects on the general population. Several studies have provided evidence that low-dose environmental exposures to EDCs are associated with disorders in humans as well as wildlife (17, 70, 211, 212). Research shows that many SHR-mediated adverse effects of EDCs may be non-linear or even non-monotonic and such effects commonly occur in dose ranges exerting no overt cytotoxicity (213–218). One of the setbacks due to these non-classical responses is in extrapolating the effect from high doses to low doses or vice versa because there is no linear relationship (219). Defining safe limits for such chemicals also becomes troublesome.

Most of the evidence for NMDR comes from cell culture and animal studies. Several epidemiological studies have also focused their attention on NMDR in EDCs recently but they differ significantly in their conclusions (23, 219). However, these differences in conclusions can arise from several inherent drawbacks in human epidemiological studies, for example, cross-sectional vs. prospective design dichotomy and difference in study analysis methods. In addition, there is a lack of sufficient knowledge about kinetics of these chemicals, their interactions with adiposity and diet, and changes to these factors with age and gender. Furthermore, in epidemiological studies, populations are often exposed to a variable mixture of chemicals at various exposure levels for various durations, and predictably, these interactions are complex. These factors make human studies difficult to interpret.

## Epigenetic and Transgenerational Effects of EDCs

More recently, scientists have discovered evidence showing that EDCs can modify gene expression without mutating DNA, which is also known as epigenetic change (220). The known possible mechanisms of epigenetic changes include methylation of cytosine residues in DNA, post-translational modification of histones and altered microRNA expression. These changes may lead to transgenerational effects on numerous organs and organ systems and are thought to be tissue-specific as well as dose-dependent (221–223). Epigenetic transgenerational inheritance is the phenomenon where environmental exposures of a woman during gestation lead to germline epimutations, which are then transmitted to subsequent generations with an observable phenotypic expression (224, 225). BPA is the most widely studied EDC in terms of epigenetic changes, and several animal studies support this theory (226–228). Other chemicals implicated in epigenetic changes leading to transgenerational effects are dioxins (229, 230), DDT (231, 232), and phthalates (233, 234). In addition, the epigenetic changes are also thought to cause latent effects in case of early life exposure, with manifestation later in life (235). However, most of these studies have evaluated only the effects on the reproductive system of experimental animals. The epigenetic changes add another dimension to understand the complexity and mechanism in the studies of environmental exposures and their effects.

## Timing of EDC Exposures as a Critical Element

EDCs influence and modify the hormonal homeostasis and, therefore, it is plausible that their effects will be more pronounced in periods during which marked hormonal changes occur, such as perinatal period, puberty and pregnancy. Tissue differentiation and maturation of several organs occur during these “critical windows” which may be influenced by EDCs (236). Moreover, the effects of exposure may not be apparent immediately and may take several years to be observable. This is supported by several studies that show the association between several health and behavioral parameters and EDC exposure during these key periods. *In utero* exposure to several EDCs was found to influence fetal growth and birth weight (109–112, 117) and also predisposed some individuals to obesity and fat gain during later life (17, 21, 22). Exposure to EDCs during the prenatal and perinatal period has been thought to influence the timing of puberty as well (86, 88). Several studies evaluating the neurological development of children have shown association of prenatal EDC exposure with adverse cognitive and behavioral outcomes (145, 146, 148, 151–153). Prenatal exposure to phthalates was shown to alter thyroid homeostasis in pregnant women as well as the offspring (167).

For POPs, latency could be due to long-term continuous exposure. For non-persistent EDCs, exposure could be variable. Apart from the molecular mechanism, epigenetic changes may be important in delayed or latent effects, especially in perinatal exposures.

## Risks From Exposure to EDCs Mixture: Cocktail Effect

EDCs do not behave in the manner of usual toxins. Unlike occupational exposure, environmental exposure to ubiquitously present contaminants occurs not only in low doses as discussed earlier, but also simultaneously to a variety of chemicals in varying combinations. Several of these EDCs act through the same receptors either in agonistic or antagonistic mechanisms. The possible combined effects may be additive, synergistic, antagonistic or nullifying (237, 238). In combination, it is even possible to affect different from individual chemicals. It has been demonstrated in animal studies that combinations of EDCs can produce effects in levels at which these chemicals do not induce any measurable effects individually (237, 239). This unique property of EDCs has been termed as “cocktail effect” by some researchers (240). The interactions of chemicals in the mixture appear to be dose-dependent, adding to the complexity further. One of the drawbacks of most epidemiological studies and animal studies is that they take into account each chemical one by one. Thus, the results in these studies may not precisely effect reflect the environmental effect. A study evaluating presence of mixtures of organochlorines in the sera of healthy women and women diagnosed with breast cancer found that only dichlorodiphenyldichloroethane (DDD) individually showed a moderate increase in the risk of developing breast cancer. They also found that a combination of aldrin, p,p′-DDE and DDD was more frequently present in breast cancer patients while it is absent in healthy women (241).

## Gutmicrobiome: A Player in the Toxicity of Environmental Pollutants

Human beings are exposed to different chemicals through air, water and soil, but oral exposure to EDCs, largely via the food chain, is their key pathway into human metabolism. The gut microbiome comprises trillions of bacteria, viruses and fungi, and it is documented as a main participant in the metabolism of nutritional compounds, drugs, antibiotics and environmental toxins (259–264). Bacterial fermentation of undigested carbohydrates and xenobiotic compounds produces short-chain fatty acids, choline, bile metabolites and several volatile compounds including hydrogen sulfide. The gut microbiome also plays a major role in regulation of various hormones, neurotransmitters and ultimately host metabolism. Some metabolites produced during bacterial fermentation in gut behave as hormones that can affect diverse host metabolic functions, and hence the gut microbiome has been anticipated to be a new endocrine organ (265–267). Dysbiosis of gut microbiome is commonly observed in medical conditions. Concerning diabetes, a shift in the *Bacteroidetes*/*Firmicutes* ratio was found to have a key link with plasma glucose concentration (268). Microbial metabolism of chemicals, as well as EDCs, by gut microbiome can lead to microbial dysbiosis, stimulation of specific bacterial genes, and altered microbial transformation of molecules (260, 268).

Previous researchers had determined how diet and host health could influence the gut microbiome composition (269–273). On the other hand, several acquired factors encountered on an everyday basis can exert extreme effects on the gut microbiome. Environmental chemicals, such as EDCs, heavy metals and air pollutants are increasingly invasive in terrestrial and aquatic environments, and there is a signal that such chemicals will turn out to be even more profuse in near future (274–281). Such chemicals are also found in things that we use daily, such as plastic water bottles, storage containers, and antibacterial supplies. Introduction to such chemicals can result in extensive host effects and concurrently aim commensal microbes harbor within the gut and perhaps other organs. The chemical-induced dysbiosis of the gut microbiome leads to disruptions in various host systems, mainly the central nervous system, via the gut–microbiome–brain axis (282–284). An improved understanding of how environmental chemical-induced gut microbiome dysbiosis changes strengthen host disease is essential. Disorders associated with gut dysbiosis comprise metabolic, neurobehavioral, immunological, cardiovascular, gastrointestinal and several other disease states (273, 285–291). The gut microbiomes have a different class of enzymes such as Phase I and Phase II. They can metabolize environmental xenobiotic chemicals and environmental chemicals may cause dysbiosis of gut microbiome, with potential effects on human health. Based on previous studies we may conclude that gut microbiome plays a major role in the toxicity of environmental pollutants.

## Evaluating Health Effects of EDCs in Humans: Is Exposomes Mapping the Key?

Breakthrough in the mapping of the human genome has laid the foundation of potent tool to classify genes and biological processes underlying any feature influenced by inheritance, including diseases. Now a similar approach can be used for mapping the “exposome”—the aggregate of environmental influences and biological responses throughout the life span (292, 293). The major challenge in EDC research is the difficulty in precisely measuring the exposure during critical sensitive windows as well as throughout the lifespan (292). Emerging technologies in genomics, epigenomics and mitochondriomics are novel opportunities to bridge the gap in exposome measurement and precision in EDC research. Every aspect of the continuously changing external and internal environment (294) constantly affects Epigenomics, proteomics and metabolomics. Extensive exposome approach will facilitate the assessment of deleterious health effects of EDCs in humans through targeted biomarkers that restructure past environmental exposure and forecast future hazard (295, 296).

## Impact of EDCs in the Context of Climate Change

Climate change has been globally acclaimed as a major environmental risk, also when combined with chemical pollution and habitat loss it has the potential to have a severe impact on human health. The impact of escalating climate change has added exigency to the rising problem of EDC's pollutants. A study by Brown et al. (297), the first of its kind, showed that climate change could amplify the deleterious effects of pollution from EDCs on aquatic environments. Climate change is influencing the rate at which toxic chemicals are released from plastic materials, stockpiles and polluted sites. Higher temperatures increase the release of persistent organic pollutants (POPs) to air by changing their rate of partitioning between air and soil, and between air, water and sediment. POPs persist in the environment, bioaccumulate through the food chain, and pose a threat of causing detrimental effects to human health (298). The fate of environmental POPs in these ecosystems is controlled mainly by temperature and biogeochemical processes. Climate change may increase the re-emissions and redistribution of POPs in different environmental settings via soil–air exchange. Soil–air exchange is a main process of controlling distribution of POPs and terrestrial ecosystem carbon at local and universal scales.

Each POP has a moving potential in air and/or water and can bioaccumulate in biological system such as lipid-rich tissues and biomagnified through terrestrial and aquatic food chains (299). Hence, POPs have a potential adverse impact on higher trophic organisms in aquatic as well as environment (300–302) and on human health (303). Moreover, intensification of global warming, an increase in POPs levels has been reported in different settings of environment because of the release from reservoirs (299, 304). These higher emissions induced by climate change would increase the susceptibility of exposed animals and humans via food chain and lead to greater adverse effects on human health and terrestrial ecosystems (305).

While the researcher's grapples with the problem of ever-increasing environmental pollution, at the same time, the causes of climate change and its impacts on the fate and toxicity of chemicals must be addressed.

## EDC Regulations in Various Countries

The issue of EDCs is puzzled due to lack of scientific data and many gaps in knowledge, it is still essential to frame regulatory policies to deal with the present scenario. Framing of policies and regulations must go parallel with ongoing research and any measure must be compliant to rapid advances in scientific knowledge. At present countries having regulations addressing the manufacture, use and disposal of various chemicals includes carcinogens, teratogens, mutagens and substances that interrupt reproduction. EDCs represent a relatively new classification, involving diverse chemical classes that are able to mimic endogenous hormones. Although there is no agreement on their regulation, EDCs are addressed in different cases in EU law, such as the Water Framework Directive, Registration, Evaluation and Authorization of Chemicals (REACH), Plant Protection Products Regulation (PPPR) and Cosmetic Regulation (Table 2). The government and regulatory agencies of developing countries with large population and huge plastic consumers may restrict the use of endocrine disrupting chemicals in the production of cosmetics, food-contact plastics, plastic water tanks and packaging materials and may provide guidelines on the use of EDC's/plastics, awareness on its harmful impacts and the use of risk-free alternatives must be provided, with an intention to ensures the safety of vulnerable group such as infants, children and pregnant women. The challenges that arise from the field of endocrine disruption are the immense diversity of chemicals produced, but not tested, the mixtures and the unknown interactions between them, together with their consequent effects. The lack of an effective and strong legislation and regulation poses a significant threat to humans, animals and plants, and contributes to the exposure to chemicals that may disrupt the endocrine system.

**Table 2:** Regulations on controlling EDCs in various countries.

**Sl. No**.	**Regulatory bodies**	**Recommendations implemented /proposed**	**References**
**1. European Union**			
1.1	European food safety authority (EFSA)	Regulations (EU) No 528 /2012 and (EC) No 1107/2009 have been developed to help assessor of the regulatory authorities on the hazard identification for endocrine disrupting properties on the scientific criteria	(306–308)
1.2	European chemicals Regulation (REACH)	ECHA's (ECHA/NR/18/36) endocrine disruptor (ED) assessment list includes the substances undergoing an ED assessment under Registration, Evaluation, Authorization and Restriction of Chemicals (REACH) For REACH substances, inclusion in the list means that an informal hazard assessment for endocrine-disrupting properties either is under development or has been completed since the start of the implementation of the SVHC Roadmap in February 2013 The legislation has been successfully implemented on the ban of BPA-containing materials of any sort which come in direct contact with food and pose threat to infants, children and pregnant and nursing mothers	(309–311)
1.3	Plant Protection Products Regulation (PPPR)	Identification of EDCs based on hazard assessment In the case of exemptions: following a risk assessment, a substance can be approved regardless of its hazards when it is necessary to control a serious danger	(312)
1.4	Biocidal Products Regulation (BPR)	Identification of an EDC based on a hazard assessment	(313)
1.5	Water Framework Directive (WDF)	The WDF deals with the development of methods for measurement of estrogens, to comply with the Water Framework Directive requirements (Directive 2013/39EC, Commission Directive 2009/90/EC and Commission Implementation It recognizes “substances and preparations, or the breakdown products of such, which have been proved to possess carcinogenic or mutagenic properties or properties which may affect steroidogenic, thyroid, reproduction or other endocrine-related functions in or via the aquatic environment” as so-called “main pollutants.” Furthermore, EDCs may be listed as priority chemicals by the EU Commission Decision (EU) 2018/840)	(314)
1.6	EU cosmetics regulation (Regulation (EC) No 1223/2009 on cosmetic products)	EU cosmetics regulation address EDCs	(315)
**2. United States**			
2.1	U.S. EPA's Safe Drinking Water Act/Toxic Substances Control Act (TSCA)/Federal Food, Drug and Cosmetic Act (FD&C Act)	The U.S. EPA's developed Endocrine Disruptor Screening Program (EDSP) that is one of the only regulatory programs designed around chemical mode of action EDSP uses two tired screening and testing strategy (EDSTAC 1998) to screens and tests environmental chemicals that have potential effects in estrogen, androgen, and thyroid hormone pathways The United States (US) started with the Toxic Substances Control Act (TSCA) specially for BPA, followed by integration of the respective EDC controls in the Food Quality Protection Act (FQPA) and the Safe Drinking Water Act (SDWA)	(316, 317)
**3. Canada**			
3.1	Canadian Environmental Protection Act (CEPA)	The Chemicals Management Plan (CMP) is a Government of Canada initiative launched in 2006 which set clear priorities for assessing and managing chemical substances used in Canada, including the new and existing substances programs of the Canadian Environmental Protection Act, 1999 (CEPA 1999).At the meeting held in July 2018, the departments sought input from the SC on scientific considerations related to how the Government of Canada could evolve the current approach for the identification and assessment of endocrine-disrupting chemicals (EDCs)	(318–321)
**4. Brazil**			
4.1	Federal Law 7802/1989	Pesticides and their components can only be approved if they are not considered to have endocrine disrupting properties	(322–324)
**5. China**			
5.1	13th 5 Year Plan of National Environmental Protection	The Ministry of Environmental Protection and the Ministry of Agriculture are taking the lead for controlling endocrine disruptors within their own jurisdictions According to China's action plan for water pollution prevention issued by the state council issued in 2015, the Chinese government plans to organize a national survey on the production and uses of Environmental Endocrine Disruptors before the end of 2017	(325)
**6. Japan**			
6.1	Japanese environmental regulation	Japan has been very active in investigating endocrine disruptors for a decade. Ministry of Environment Protection (MEP) in 1998 started Strategic Programs on Environmental Endocrine Disruptors (SPEED) with a focus on screening of environmental endocrine disruptors Japan MEP is leading a program named EXTEND (Extended Tasks on Endocrine Disruption) 2010, aiming to accelerate the establishment and implementation of assessment methodologies toward the goal to properly assess the environmental risk of endocrine disrupting effects of chemical substances	(326)
**7. South Korea**			
7.1	Korean Regulation on the Registration and Evaluation of Chemicals (K-REACH)	Substances are evaluated for potential risks. EDCs can be recognized as substances subject to authorization, restriction or prohibition	(327)
**8. Australia**			
8.1	National Industrial Chemicals Notification and Assessment Scheme (NICNAS)	EDCs are clearly addressed under the estimation framework of industrial chemicals, the National Industrial Chemicals Notification and Assessment Scheme (NICNAS) NICNAS also provides the authority to its Director to identify and assess substances on the AICS (i.e., existing chemical substances) for their human health-related or environmental risks under the Integrated Multi-tiered Assessment and Prioritization (IMAP) framework Tier-1 assessment of IMAP defines a substance as an EDC based on the list of priority substances developed under the EU-Strategy for endocrine disruptors	(328)
8.2	Australian Drinking Water Guidelines 2011 (ADWG)	The Australian Drinking Water Guidelines 2011 (ADWG) have been revised and updated recently, incorporating guidelines for EDCs in drinking water, because of the frequent detection of EDCs in drinking water and the high incidence of illnesses associated with them, particularly among poor families	(329)
8.3	Australian Food and Grocery Council	Australian Food and Grocery Council also extended support toward reduction of BPA usage and availability of BPA alternatives	(330)
**9. New Zealand**			
9.1	New Zealand Food and Grocery Council also voluntarily involved itself in phasing-out of BPA used in polycarbonate baby feeding bottles	New Zealand Food and Grocery Council also voluntarily involved itself in phasing-out of BPA used in polycarbonate baby feeding bottles New Zealand government also advised public to take BPA alternatives until conclusive evidence against safety of PC baby feeding bottles is ensured	(331, 332)
**10. Sweden**			
10.1	Government of Sweden	The Swedish Chemical agency, had proposed a thorough investigation in 2012 on the use of BPA in thermal papers • The agency is working in cooperation with the Swedish National Board of Housing, Building and Planning and also the National Food Agency to investigate the extent of BPA migration by the use of epoxy lining in water pipelines, toys and articles used by children • In 2015, The Government of Sweden declared a ban on the use of BPA in food packaging materials for children under the age of three • Concerning the risks of negative effects on infants exposed to BPA in tap water, the Swedish government proposed to the European Union to restrict BPA in relining of pipelines	(333)

## Conclusions

Most of the information on the detrimental effect of EDCs comes from animal studies. It is becoming more evident that EDCs may trigger disorders such as metabolic diseases, reproductive abnormalities, endocrine dysfunction and cancers. The sudden escalating rates of these diseases, especially metabolic disorders, correlate with global industrialization and the production and release of EDCs to the environment, drinking water and eventually to food chain. Countries should formulate acts and guidelines including EDCs for the safety of drinking water. To understand the exposure routes of EDCs better, a comprehensive assessment of drinking water supply is required. Exposure to EDC or mixtures, even at low doses, especially during critical window or early development can have an impact on endocrine system by altering transgenerational/epigenetic pathway. Hence, further studies are required to establish the threshold concentrations of EDCs in the environment as well as in human matrixes (bio monitoring) below which its detrimental effects do not occur. Large-scale systematic epidemiology studies need to be carried out taking into consideration the low dose effect and cocktail effect of EDCs.

## Author Contributions

MKumar, MKumawat, SS, and DS: manuscript draft. RT and AP: overall review. VV: manuscript drafting and editing. All authors contributed to the article and approved the submitted version.

## Conflict of Interest

The authors declare that the research was conducted in the absence of any commercial or financial relationships that could be construed as a potential conflict of interest.
